# Group psychotherapy for patients with first-episode psychosis: Effect on the clinical status and use of resources

**DOI:** 10.1192/j.eurpsy.2023.1322

**Published:** 2023-07-19

**Authors:** P. Herrero Ortega, A. Oliva Lozano, J. Garde González, C. Bayón-Pérez, R. Mediavilla, M. P. Vidal-Villegas, B. Rodríguez-Vega, S. Cebolla, E. Román, E. V. Pérez Pérez, M. F. Bravo-Ortiz, O. B. O. AGES-Mind Group

**Affiliations:** 1 Department of Psychiatry, Clinical Psychology and Mental Health, La Paz University Hospital; 2 School of Medicine, Autonomous University of Madrid (UAM); 3 Hospital La Paz Institute for Health Research (IdiPAZ); 4 La Paz University Hospital Biomedical Research Foundation (FIBHULP); 5 Centro de Investigación Biomédica en Red de Salud Mental (CIBERSAM), Madrid, Spain

## Abstract

**Introduction:**

Psychotic disorders carry several economical, psychological and social consequences, both at individual and community levels. Early intervention programs after first-episode psychosis which combine pharmacological and psychosocial strategies are aimed at reducing symptoms, lowering costs in the use of health and non-health care resources and improving overall functioning. AGES-Mind study is based on manualized psychotherapeutic interventions for people with first-psychosis episodes.

**Objectives:**

The aim of the study was to evaluate the effect of a group psychotherapeutic intervention on the clinical status and use of clinical resources in a sample of patients with first-episode psychosis at 12 and 24 months after the beginning of the intervention. This cohort will be compared to patients with first-psychosis episodes without group psychotherapeutic intervention.

**Methods:**

Longitudinal, observational, retrospective study on a cohort of N=46 patients with first-episode psychosis within the last 5 years. Two groups of 23 patients each were formed. The participants of one of those groups received group psychotherapy in the context of the AGES-Mind study and the other group received treatment as usual without group intervention. Non-exposed patients were matched by age, gender and time elapsed since first-episode psychosis with those exposed to the intervention. Sociodemographic data, clinical status and use of clinical resources outcome variables were assessed.

**Results:**

No significant differences were found in clinical status and use of resources between participants and non-participants in the psychotherapeutic group intervention after 12 and 24 months.

**Conclusions:**

After controlling for potentially confounding variables as sociodemographic, age and time since first-episode, participating in a group psychotherapeutic program does not seem to improve clinical variables or use of resources. Further studies with larger samples would be necessary to explore other variables, such as symptoms, satisfaction with the intervention or social functioning.

**Disclosure of Interest:**

None Declared

